# Behavioral Drivers Influencing Women’s Decision to Use Self-Injectable Contraception Provided by Community Health Surveillance Assistants in Rural Malawi

**DOI:** 10.1089/whr.2025.0022

**Published:** 2025-05-19

**Authors:** Martha Kamanga, Dilys Walker, Address Malata, Mandaachepa Nyando, Jessie Salamba, Alaizi Nkhoma, Innocencia Mtalimanja, Tamanda Jumbe, Emas Potolani, Alfred Maluwa, Chifundo Zimba, Josophine Changole, Rabecca Bika, Emily Himes, Lauren Suchman, Janelli Vallin, Beth Phillips, Jenny Liu, Kelsey Holt

**Affiliations:** ^1^ Department of Midwifery, School of Maternal, Neonatal and Reproductive Health, Kamuzu University of Health Sciences, Lilongwe, Malawi.; ^2^ Department of Obstetrics, Gynecology and Reproductive Sciences, School of Medicine, UCSF, San Francisco, California, USA.; ^3^ Malawi University of Science and Technology, Academy of Medical Sciences, Limbe, Malawi.; ^4^ School of Nursing, Kamuzu University of Health Sciences, Lilongwe, Malawi.; ^5^ Reproductive Health Directorate, Government of Malawi Ministry of Health, Lilongwe, Malawi.; ^6^ Nursing and Midwifery Department, Government of Malawi Ministry of Health, Lilongwe, Malawi.; ^7^ Department of Family and Community Medicine, School of Medicine, UCSF, San Francisco, California, USA.; ^8^ Department of Social and Behavioral Sciences, Institute for Health and Aging, School of Nursing, UCSF, San Francisco, California, USA.; ^9^ Institute for Health and Aging, School of Nursing, UCSF, San Francisco, California, USA.

**Keywords:** behavioral drivers, use self-injectable contraception, women’s decision making, community health surveillance assistants

## Abstract

**Introduction:**

Self-injection (SI) for contraceptive use is recommended for its proven ability to empower women and overcome barriers to contraceptive access. The World Health Organization endorsed SI as a self-care approach in 2019. Despite the increase in Malawi’s modern contraceptive prevalence rate from 38.1% in 2012 to 48.9% in 2020, it remains below the government’s 60% target. Injectable contraceptives, including depot medroxyprogesterone acetate subcutaneous (DMPA-SC), introduced in 2018, are the most popular contraceptive method in Malawi, particularly among adolescents, representing 49.8% of the contraceptive method mix. However, utilization of SI remains limited, especially in rural areas where access challenges persist. This study explores the behavioral drivers influencing women’s decision to use self-injectable contraception provided by community health surveillance assistants (CHSA) in rural Malawi.

**Methods:**

Using the capability, opportunity, motivation—behavior model, the study analyzed drivers of DMPA-SC SI adoption among 60 women aged 15–45 years in two rural districts, Mulanje and Ntchisi. Data were collected through in-depth interviews on women’s experiences with contraceptives, including SI.

**Results:**

Women's capability was strengthened as CHSAs addressed initial hesitation through practical demonstrations. Opportunity improved through enhanced access, trust, and privacy. Motivation increased with counseling, reduced travel costs, and CHSAs’ support, encouraging women to adopt self-injection confidently and consistently.

**Discussion:**

Women’s decisions to adopt SI were shaped by capability, motivation, and opportunity, with CHSAs playing a pivotal role. Future family planning programs should prioritize CHSAs’ training and deployment to improve SI uptake, fostering autonomy and accessibility for rural women.

## Introduction

The self-injectable contraceptive, depot medroxyprogesterone acetate subcutaneous (DMPA-SC), has gained significant attention for its potential to empower women and enable contraceptive usage among women who desire pregnancy prevention. The World Health Organization in 2019 recommended self-injection (SI) as a self-care approach, advocating its integration into standard contraceptive care as an additional method for delivering injectable contraception to women.^1^ This approach aligns with the broader self-care movement, which aims to enhance people’s active participation in their own health care and promote their autonomy and engagement in their own well-being.^2^

Training community health surveillance assistants (CHSAs) is crucial for successfully implementing self-care initiatives such as DMPA-SC SI in Malawi, particularly in rural areas. As frontline health workers, CHSAs are often the primary point of contact between women and health care services, making their role vital in educating, motivating, and supporting women to adopt new contraceptive methods that align with women’s contraceptive preferences. They play a crucial role in expanding access to contraceptive services and educating clients in Malawi. Evidence shows that CHSAs have been instrumental in increasing contraceptive uptake, particularly through community-based distribution of injectables such as DMPA-SC. A study by FHI 360 found that CHSAs, the local cadre of CHSAs, effectively attracted new family planning clients and increased access to contraceptives in rural areas.^3^ In addition, a systematic review found that ∼93% of CHSAs-led family planning programs resulted in increased modern contraceptive use, while 83% improved knowledge and attitudes toward contraception.^3^ Given their trusted position within communities, CHSAs play a key role in educating, motivating, and supporting women to adopt contraceptive methods that align with their preferences, ultimately enhancing reproductive health outcomes. Comprehensive training should equip CHSAs with the necessary knowledge, skills, and confidence to provide accurate information, demonstrate SI techniques, and address any concerns or misconceptions women may have while respecting women’s right to autonomous, informed decision making.^4^ Moreover, contextual challenges in Malawi, such as geographical distances, prolonged clinic waiting times, and intermittent shortages of essential commodities, hinder certain women from accessing their preferred contraceptive methods.^3^

In Malawi, the government aims to increase the use of contraception among women who do not desire to be pregnant by enhancing access to reliable modern contraceptive methods.^5^ The country has made strides in increasing contraception prevalence in Malawi, from 38% in 2012 to 49% in 2020,^6^ close to the 60% modern contraceptive prevalence rate target set by the Government Family Planning Commitment (2020).^5,7^ The introduction of DMPA-SC SI in Malawi in 2018 was a crucial step in expanding the contraceptive method mix and generated hope that this self-care option could help additional women use contraception when they desire pregnancy prevention. The country’s commitment to SI emerged from the success of a randomized controlled trial of DMPA-SC conducted from 2015 to 2017 in one district.^8^ This randomized controlled trial revealed that women who self-injected DMPA-SC had significantly higher continuation rates (73%) compared with those who received provider-administered injections (45%), with an incidence rate ratio of 0.40 (95% confidence interval [CI]: 0.31–0.51; *p* < 0.0001). Following the promising results of this trial, the Malawi Ministry of Health initiated a rapid scale-up of DMPA-SC SI in 2018, ∼8 months after the trial concluded, including training of CHSAs to provide DMPA-SC for SI. However, despite the initial momentum, in the fourth quarter of 2020, only 12% of new injectable users opted for the SI method, contrasting with earlier acceptability studies that suggested higher potential uptake rates.^5^

While prior research done in rural Mangochi District of Malawi and elsewhere demonstrates the acceptability of SI,^3^ there remains a lack of insight into the motivations and barriers influencing women’s decisions regarding SI outside of controlled environments. Our team’s four-country study of perceptions of SI in sub-Saharan Africa revealed that women see value in the privacy, convenience, and control that SI offers.^9^ At the same time, they recognize limitations to these benefits, such as having to store doses in one’s home or travel to a health service for initial training.^9^ Yet, little has been published on specific behavioral drivers shaping women’s choices to use or not use SI in everyday life. Data are sparse on whether the gap in SI utilization reflects women’s actual preferences or if there is a programmatic shortfall that could help interested women take advantage of SI. In understanding the dynamics of SI as a contraceptive option in Malawi, it is crucial to acknowledge that all women do not universally prefer SI. While some may find it suitable, others may not, underscoring the importance of exploring the reasons behind nonutilization despite availability.

CHSAs typically undergo a standardized training program that equips them with the skills to deliver essential healthcare services, including family planning, health education, and disease surveillance. On average, each CHSA serves a population of ∼1,000–2,000 people, making them a crucial link between the health care system and underserved populations. Despite CHSAs’ accessibility and efforts to promote SI, women’s decisions to adopt this method may be influenced by factors such as limited contraceptive knowledge or low self-efficacy—both areas that could benefit from targeted programmatic interventions. To investigate this further, our study sought to answer the question: What behavioral drivers influence women’s decisions regarding the use of DMPA-SC SI for self-care when the method is offered by CHSAs?

## Materials and Methods

### Theoretical framework

The capability, opportunity, motivation—behavior (COM-B) change model guided this study. COM-B outlines the drivers behind health-related behaviors and sheds light on the motivations that influence contraceptive choices and the decision to use or continue using DMPA-SC SI. This model positions the interaction of COM as catalysts for behavioral performance (B), offering potential insights into the reasons behind nonengagement in new contraceptive options.^10^

The data for this article were collected as part of the Innovations for Choice and Autonomy (ICAN) study, a collaborative effort involving research institutions in the United States and sub-Saharan Africa. The ICAN consortium includes partners at the Malawi University of Science and Technology (MUST), Makerere University School of Public Health in Uganda, Kenya Medical Research Institute in Kenya, Akena+ Health in Nigeria, and the University of California, San Francisco in the United States. The overarching goal of ICAN is to explore the implementation of SI of DMPA-SC to meet women’s needs best, as defined by women themselves.

### Study design

We used a qualitative cross-sectional approach to conduct in-depth interviews with 60 women in two rural districts of Malawi, namely, Mulanje and Ntchisi. Using the COM-B model, the study analyzed drivers of DMPA-SC SI adoption among 60 women of reproductive age.

### Study population

A total of 60 women within the reproductive age group of 15–45 years participated, divided into two categories: those who had never used contraceptives and those who had prior contraceptive experience. Each category was further stratified into subgroups aged 15–19 and 20–45 years to explore age-related differences.

Women were purposively sampled to ensure diversity of backgrounds and experiences, focusing on age, prior contraceptive use, and previous experience with DMPA-SC. Potential participants were identified through collaboration with CHSAs, who maintain regular contact with women in their communities and were instrumental in referring eligible participants to the study team. While this recruitment approach enabled access to a broad spectrum of perspectives and facilitated participant trust and engagement, it may have introduced selection bias. Women with limited or no contact with CHSAs were likely underrepresented, potentially excluding views from those who face challenges in accessing community-based contraceptive information and services. This limitation is important when interpreting findings related to CHSA effectiveness and accessibility, and it is further addressed in the discussion section under limitations.

#### Inclusion criteria

Eligible participants included women aged 15–45 years who were sexually active, regardless of their marital status. They needed to speak either Chichewa or English. In addition, all participants were required to have the ability to provide written informed consent for their involvement in the study.

#### Exclusion criteria

Women under 15 or over 45 were deemed ineligible for inclusion. This approach was taken to ensure that participants could provide meaningful insights into contraceptive use, as the perspectives of individuals who are not sexually active or are beyond the reproductive age might offer limited relevance to the study’s objectives.

### Data collection method

In-depth interviews were conducted in the Chichewa language using semistructured interview guides from March to April 2021. The interview guide included questions about women’s experiences and decisions related to contraception, including DMPA-SC for SI. The interviews were audio-recorded, and field notes were taken to capture nonverbal cues and observations during the interviews. Interviews lasted ∼30–45 minutes.

### Data analysis

Interview recordings were transcribed verbatim, translated into English, and imported into Dedoose software for qualitative analysis. We created the codebook using a mix of the guiding framework from the guides and codes developed inductively as we reviewed the data. Data were analyzed using thematic analysis, which involved the steps of data familiarization, coding, theme generation, and validation. The analysis was guided by the COM-B model, which provided a structured framework to identify specific drivers influencing women’s use or nonuse of SI. Expert analysts reviewed the data to ensure that the identified themes accurately captured the behavioral determinants. The detailed ICAN qualitative analysis process is described comprehensively in Suchman et al. (2023).^11^

The transcripts were de-identified to protect participants’ privacy, and all personal details were removed. Access to audio recordings and coded transcription files was restricted to authorized study investigators and designated study personnel, ensuring strict confidentiality throughout the research process.

### Ethical considerations

Ethical approval for the research was obtained from the UCSF Institutional Review Board (270555, 270747, 270554, 270084) and the MUST Research Ethics Committee (P.03/2020/007).

We obtained informed consent for participants who were legally authorized to provide informed consent or assent for minors who could provide their agreement. Parental consent was exempted for minors considered emancipated, meaning those who are legally recognized as independent from parental or guardian control due to specific circumstances such as marriage in this scenario. This exemption was made to prevent barriers to participation, as requiring parental consent in such cases could exclude otherwise eligible women from participating in the research. It could also disclose the subject’s current/past sexual activity and/or use of or interest in contraception to her parents, thus violating the subject’s confidentiality.

All interviews were conducted in a private, confidential space identified by the participants to protect the privacy of women and adolescents.

## Results

### Demographic characteristics

The study sample consisted of 60 women aged between 15 and 45 years. Of these, 18 (30%) were adolescents aged 15–19 years, while 42 (70%) were aged 20–45 years (Table 1). Most participants were married and had children under the age of 5.

**Table 1.** Participant Characteristics (*N* = 60)

Characteristics	*n*	%
Age		
15–19	18	30%
20–45	42	70%
Contraceptive use history		
Never used	22	37%
Prior or current use^a^	38	63%
DMPA-SC history		
Never used	30	50%
Ever used DMPA-SC	30	50%
Ever self-injected DMPA-SC	18	30%
Parity^b^		
Nulliparous	16	27%
Ever had children	43	72%
Marital status		
Married	40	67%
Unmarried	19	32%
Partnered/cohabitating	0	0%
Married, husband has multiple wives	1	2%

^a^ Contraceptive use was defined as using any effective method of pregnancy prevention, including so-called “traditional” methods such as withdrawal or fertility awareness methods.

^b^ Missing data from one participant.

DMPA-SC, depot medroxyprogesterone acetate subcutaneous.

On contraceptive use history, 22 women (37%) had never used any contraceptive method: 13 from the 15–19 age group and 9 from the 20–45 age group. Among this group of noncontraceptive use, the study did not systematically assess whether these women had previously received contraceptive counseling, been offered contraception, or faced access barriers. This represents a key limitation, as such information could have provided important insights into reasons for nonuse and potential gaps in CHSA engagement or health system support.

The remaining 38 women (63%) had a history of contraceptive use, comprising 8 adolescents and 30 adults. Of these, 18 women (30% of the total sample) were current users of DMPA-SC through SI at the time of data collection. The other 20 participants included women who were using alternative contraceptive methods (such as provider-administered injectables, oral contraceptives, or implants), as well as some who had previously used DMPA-SC SI but had since discontinued or were not using any method at the time.

Although the study was not designed to systematically quantify switching or discontinuation patterns, participant narratives revealed several influencing factors. These included preferences for provider-administered methods, fear of incorrect SI, stockouts of DMPA-SC, and concerns about side effects. These findings highlight the need for deeper, structured inquiry into women’s contraceptive trajectories, including reasons for discontinuation and the role CHSAs can play in addressing both the adoption and sustained use of SI methods.

### Behavioral drivers that influence women’s choice to use DMPA SC SI in the context of CHSA-provided contraception services

**FIG. 1.**
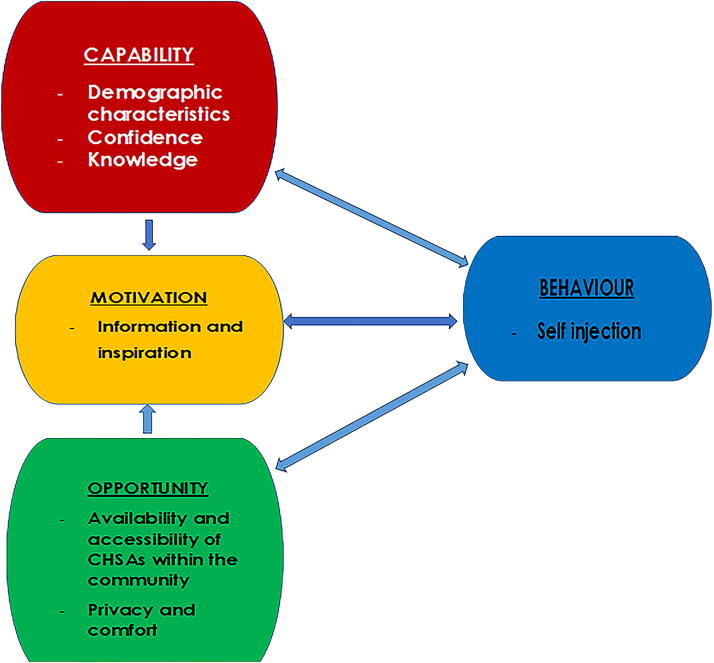
Factors influencing women’s decisions to use or not use depot medroxyprogesterone acetate subcutaneous self-injection.

Employing the COM-B change model, our analysis focused on identifying the key factors driving women’s choices, namely capability, motivation, and opportunity, which may be influenced by the health service environment—in particular, their interactions with frontline CHSAs who provide the majority of contraceptive care in the two rural study districts. Our study findings indicate that women’s decisions regarding the use or nonuse of SI are influenced by many complex factors related to their capability, motivation, and opportunity. These factors are influenced by their interactions with CHSAs, as described below and summarized in Figure 1.

#### Capability

We identified a lack of confidence and limited knowledge related to SI as common drivers of nonuse of SI. Among users of SI, detailed information from CHSAs was a key factor in their decision to use the method.

##### Confidence

Many women who described being apprehensive about SI lacked confidence in their ability to administer medication to themselves. As one participant described:

If it scares me to get injected by someone, how about doing it by myself on my body? No, I cannot do that. *(NS 020, married, never used contraception)*

Among those using SI, many described a sense of control that motivated them to continue using the method. For example, one participant noted:

I inject myself very slowly and calmly until I notice that now I can start pressing the drug in my body. … I have control over myself and power because I can do it in my free time while I am done with other chores. *(NS 09, in a polygamous relationship, DMPA-SC SI user)*

##### Knowledge

When women without previous experience with SI were asked about their knowledge of the method, many demonstrated limited awareness or misconceptions. For instance, one respondent shared:

Some contraceptive methods don’t interact well in the body, like having heavy continuous menses, that means you have to switch methods so I feel that these things are difficult because you can do family planning on your own and prevent pregnancy, so I feel that I should just be doing these things on my own without involving the government and their methods. *(MJ 028, married, never used contraception)*

Those who CHSAs had trained in how to self-inject described appreciating the variety of teaching aids and methods CHSAs used, including hands-on demonstrations with the DMPA-SC injection vial and visual aids such as posters, picture cards, and projected images. Many emphasized the importance of the knowledge they gained from CHSAs that influenced them to begin using SI. As one participant expressed:

I went there, I found the community HSA and he gave me what I wanted, and I was so happy about it. He showed us how we would be doing the self-injection, so I was very satisfied with the way he demonstrated. He used the SAYANA device to demonstrate how we should do the self-injecting. *(NS 02, married, DMPA-SC SI user)*

#### Opportunity

##### Availability and accessibility of CHSAs within the community

The physical presence of CHSAs near women’s homes was highly valued by interviewees, who stressed the critical role this played in their ability to access DMPA-SC SI and other contraceptive methods. One participant noted:

We have a provider (HSA) in our community, every community has a Health Surveillance assistant. … The provider explained to us about DMPA SC SI before it got here … he is the only one who lives close to us, so it’s very easy to communicate with him. *(MJ 01, married, DMPA-SC SI user)*

This highlights how CHSAs’ proximity facilitates communication and education about SI, thus increasing the likelihood that women will try this method.

Moreover, another woman echoed this sentiment, emphasizing the accessibility of CHSAs in times of need: “When you have a concern, you go to find them, even though during the night you got sick or you are experiencing any issues with contraceptive you also go to find the community HSA to help you” (MJ 08, married, DMPA-SC SI user). This highlights the continuous support provided by CHSAs, especially during emergencies or concerns related to contraceptive use.

While many participants valued CHSAs’ role in supporting DMPA-SC SI, some interviewees highlighted that their local CHSA needed training in the method, which inhibited their ability to fully assist women interested in SI. In addition, stockouts posed a significant barrier, limiting the CHSAs’ ability to provide timely support. One participant emphasized the importance of both training and a reliable supply:

… the only support I will need is that the community HSAs should also be supplied with this injection method that we inject ourselves. The reason is that someday it can happen that you are very occupied, either you are far, or you have forgotten that you were supposed to go to the hospital to get family planning method. You are just remembering in the evening around past five and by that time they have already closed the hospital, you can just go to the community HSA even during the night like past 8. This means that they can give you, and you will be able to inject yourself without any problems. *(MJ 03, married, DMPA-SC SI user)*

This highlights the dual challenge of training gaps and stockouts, which affect CHSAs’ ability to support women seeking SI. It also underscores the convenience of CHSA-based provision, particularly for women facing logistical barriers to accessing health facilities.

#### Motivation

##### Information and Inspiration

Women who had adopted SI described the instrumental role CHSAs played in informing and motivating them to choose self-injectable contraceptives. The CHSAs’ comprehensive knowledge and approachable conduct significantly influenced women’s decisions. For instance, one woman mentioned:

The HSAs have adequate information related to DMPA SC SI. To us, DMPA-SC SI is new, and we know more about Depo Provera compared to DMPA-SC SI. … I do rely more on CHSAs, and for CHSAs located in our area, they can help me in deciding contraceptives, including DMPA-SC SI, they are open and they cannot refuse to help. *(NS 025, married, DMPA-SC SI user)*

The women observed that the information and counseling provided by CHSAs played a crucial role in motivating them to consider and adopt SI. One woman described how the CHSA introduced her to the self-injectable method, DMPA-SC SI, highlighting the financial benefits: “I felt this method is good because it will help me save my transport money. I decided to start using it and was told I will be given three doses of DMPA-SC SI” (MJ 10, married, DMPA-SC SI user), reflecting how CHSAs effectively communicated the advantages of SI, particularly in terms of cost savings and convenience, which resonated with women facing economic and logistical barriers.

Another woman emphasized the significant role of the CHSA, noting that beyond counseling, their accessibility made SI more convenient: “There is a long distance between where I stay and the hospital, so I spend a lot on transport. When I received contraceptive counseling from the CHSAs, they introduced me to DMPA-SC SI. It was a good method because I would save on transport money and not need to visit the hospital frequently. Now, when it’s time for DMPA-SC SI, I inject myself at home” (NS 05, married, DMPA-SC SI user), illustrating that the detailed and personalized information provided by CHSAs not only informed women about SI but also empowered them to overcome challenges associated with accessing contraceptive services, thereby increasing their motivation to adopt and continue using SI.

## Discussion

Our findings shed light on the complex behavioral drivers influencing women’s decisions to use DMPA-SC SI in the context of contraceptive services provided by CHSAs in Malawi. By applying the COM-B model, which considers capability, opportunity, and motivation as central factors, we gained a deeper understanding of women’s contraceptive decisions and the vital role CHSAs play as critical frontline health workers in rural Malawi. These findings align with global evidence on the role of community health workers in improving contraceptive access and uptake. Our findings shed light on the complex behavioral drivers influencing women’s decisions to use DMPA-SC SI in the context of contraceptive services provided by CHSAs in Malawi. By applying the COM-B model, which considers capability, opportunity, and motivation as central factors, we gained a deeper understanding of women’s contraceptive choices and the vital role CHSAs play as critical frontline health workers in rural Malawi. These findings align with global evidence on the role of community health workers in expanding access to contraceptives, particularly in low-resource settings where they help bridge gaps in facility-based services.^12,13^ Studies have shown that community health workers improve contraceptive uptake by increasing accessibility, providing tailored counseling, and addressing barriers to facility-based care.^14^

The initial apprehension many women felt toward SI was primarily attributed to a lack of confidence in their ability to administer injections. This barrier highlights the importance of self-efficacy, a central component of the COM-B model and a recurring theme in health behavior change literature.^15^ Studies from Uganda and Senegal show that initial apprehension is common among new users of DMPA-SC SI but can be addressed through targeted support.^16,17^ CHSAs in our research provided practical demonstrations and hands-on support, significantly increasing women’s confidence. Similar to findings in Kenya, experiential learning reinforced women’s belief in their ability to perform SI effectively.^18^ As women successfully self-injected under supervision, they experienced a sense of accomplishment, which enhanced their autonomy and control over reproductive health decisions. This empowerment, derived from mastery experiences, aligns strongly with the COM-B framework’s emphasis on capability as a driver of behavior.

Knowledge acquisition was another critical factor. The education provided by CHSAs—using visual aids and repetitive hands-on demonstrations—ensured women had the skills and understanding necessary for accurate and confident SI. This aligns with the health belief model, which posits that perceived knowledge and skill reduce barriers to health behaviors^18^ and further reinforces the COM-B model’s assertion that skills and knowledge are foundational for behavior change.

The proximity and availability of CHSAs were instrumental in facilitating access to SI and providing ongoing guidance. The importance of CHSAs as a reliable and accessible resource mirrors findings in global studies, such as those from Uganda, where the integration of community health workers into contraceptive programs enhanced user confidence and uptake.^16^ CHSAs’ presence within the community created a trusted support system that reassured women about managing potential challenges independently, further enhancing their perceived behavioral control.

While our findings highlight the critical role of CHSAs, the absence of trained CHSAs in some communities did not emerge as a widespread barrier in the formative data. Rather, some participants—particularly those already using SI—expressed concerns about the availability of CHSAs for continued support and method resupply. A more consistent challenge for nonusers appeared to be a lack of awareness or trust in the method, rather than direct limitations due to untrained CHSAs. Given similar programmatic gaps observed in Kenyan contexts, where the uneven distribution of community health workers affected program effectiveness,^18^ addressing CHSA coverage could still enhance equity in access. Future efforts should explore whether training gaps significantly impact potential users’ ability to adopt SI, particularly among those who have yet to try the method.

Privacy and comfort during interactions with CHSAs were also valued, as these factors fostered trust and open communication. Similar findings in Uganda and Senegal suggest that creating safe and supportive environments is integral to successfully adopting SI.^19^ These conditions enabled women to express their concerns openly and receive tailored advice, an approach aligned with the self-determination theory, highlighting the importance of autonomy-supportive environments in fostering intrinsic motivation.^20^

Women’s motivation to adopt SI was strongly influenced by the detailed information and encouragement provided by CHSAs. Economic benefits, such as reduced travel costs and the convenience of home administration, emerged as key motivators, aligning with findings from similar studies in Uganda, where cost savings were a significant driver of DMPA-SC SI uptake.^16^ In addition, the counseling techniques employed by CHSAs, including personalized guidance and autonomy support, reflect best practices in motivational interviewing and align with the COM-B model’s focus on intrinsic motivation as a driver of behavior.

By integrating insights from global evidence with the COM-B framework, our findings highlight the critical role of CHSAs in addressing capability, opportunity, and motivation barriers to SI adoption. Strengthening training programs for CHSAs and addressing coverage gaps represent key opportunities for scaling up and sustaining SI programs in Malawi and similar settings.

## Conclusions

The success of DMPA-SC SI adoption centers on the interplay of capability, motivation, and opportunity, heavily influenced by CHSAs. CHSAs play a vital role in building confidence, providing education, ensuring accessibility, and motivating women to use SI. Comprehensive training for CHSAs is essential, focusing on practical demonstrations, visual aids, and motivational interviewing to enhance women’s confidence and autonomy. Integrating behavior change theories into health interventions can address barriers and motivators in contraceptive use. In addition, emphasizing economic and convenience benefits, alongside opportunities for supervised practice, can further support more women in choosing this underutilized self-care technology.

## Study Limitation

This study has several limitations. First, it was conducted in selected communities, which may limit generalizability to other regions of Malawi with different sociocultural contexts, health infrastructure, and CHSA presence.

Second, the study focused on short-term outcomes related to DMPA-SC SI uptake and did not assess long-term continuation or sustainability. Variability in CHSA training and performance, which was not systematically assessed, may also have influenced the quality and consistency of support provided.

Third, the recruitment approach, which relied on CHSA referrals, may have introduced selection bias by overrepresenting women with better CHSA access or more positive experiences. This potentially excludes harder-to-reach women and limits broader applicability.

Fourth, while the study explored experiences of current DMPA-SC SI users, it did not systematically categorize contraceptive trajectories among all women with prior contraceptive use. Reasons for method-switching or discontinuation—such as side effects, provider preference, or access issues—were noted anecdotally but not formally captured. Future studies should consider longitudinal or mixed-method designs to better understand continuation patterns.

In addition, among nonusers of contraception, the study did not explore whether they had received counseling or faced barriers to access—limiting insight into reasons for nonuse and CHSA engagement gaps.

Last, while CHSA gender was not a central theme, the predominance of male CHSAs in rural sites may influence client comfort, especially among adolescents. Future studies should examine the impact of gender dynamics on contraceptive access and decision making.

## Authors' Conributions

M.K. led the conceptualization, data analysis, visualization, and drafting of the original manuscript. D.W. provided guidance on the application of the conceptual framework to the study. K.H., A.M., B.P., J.V., M.N., J.L., and A.M. played a key role in critiquing and editing the manuscript. J.S. and A.N. offered technical insight on DMPA-SC self-injection. I.M., T.J., C.Z., J.C., and R.B. contributed to the literature review. E.H. and L.S. supported the refinement of the manuscript, while E.M. was instrumental in shaping the human-centered design aspects of the study.

## Abbreviations Used

CHSA: Community Health Surveillance Assistants
COM-B: Capability, Opportunity, Motivation—behavior
DMPA-SC: Depot medroxyprogesterone acetate subcutaneous
DMPA-SC SI: Depot medroxyprogesterone acetate subcutaneous self injection
ICAN: Innovations for Choice and Autonomy
MUST: Malawi University of Science and Technology
PhD: Doctor of Philosophy
SI: Self injection
UCSF: University of California, San Francisco
USA: United States of America
